# Cost-Effectiveness of Intranasal Live-Attenuated Influenza Vaccine for Children: A Systematic Review

**DOI:** 10.3390/vaccines10091466

**Published:** 2022-09-04

**Authors:** Kenneth Sik-Kwan Chan, Charlene Hoi-Lam Wong, Horace Cheuk-Wai Choi

**Affiliations:** 1 Department of Clinical Oncology, School of Clinical Medicine, Li Ka Shing Faculty of Medicine, The University of Hong Kong, Hong Kong SAR, China; 2 School of Public Health, Li Ka Shing Faculty of Medicine, The University of Hong Kong, Hong Kong SAR, China; 3 Laboratory of Data Discovery for Health Limited (D24H), Hong Kong Science Park, Hong Kong SAR, China

**Keywords:** live-attenuated influenza vaccine (LAIV), economic evaluation, pediatric vaccination

## Abstract

Introduction: The public health burden of seasonal influenza is significant, and influenza vaccination is the most effective preventive strategy. Nonetheless, the recommendation of influenza immunization in the pediatric population is still underrepresented. Our work aimed to assess the cost-effectiveness of pediatric influenza vaccination with the intranasal live-attenuated influenza vaccine (LAIV). Methods: We performed a systematic review of publications from PubMed/MEDLINE, Embase, and Scopus, covering the period from 1 January 2000 to 30 April 2022. We searched for economic evaluations that studied the impacts of LAIV among children or the pediatric population. Studies that considered incremental cost-effectiveness ratios (ICERs), in terms of cost per gain in life years, quality adjusted life years, or disability-adjusted life years, were covered. The Consensus Health Economic Criteria (CHEC) Extended Checklist was adopted to check the quality of the included studies. Results: Thirteen studies were included for the final review that were of good or excellent quality. The implementation of influenza vaccination with intranasal LAIV in the pediatric population was cost-effective when compared to the immunization strategies for the elderly and the high-risk groups alone or with no vaccination. The efficacy of LAIV for children, vaccination coverage, and the vaccine price were significant factors to the cost-effectiveness of influenza vaccination for children. Another significant contribution to the cost-effectiveness was the herd immunity arising from pediatric immunization against influenza. Conclusions: The implementation of influenza vaccination in the pediatric population with LAIV is cost-effective. Policymakers and health authorities may consider the evidence on the development of the pediatric influenza vaccination in their immunization schedules.

## 1. Introduction

Seasonal influenza is a major public health problem worldwide due to its transmissibility and antigenic variability of viruses. It is estimated that there are one billion influenza cases per year globally, of which three to five million are severe, resulting in approximately 290,000–650,000 influenza-linked respiratory deaths [1,2]. In addition to clinical and public health impacts, the economic burden of influenza is substantial including direct health care costs and indirect societal costs associated with influenza morbidity and mortality as well as the loss of productivity [3,4]. Previous studies have demonstrated that influenza, particularly in the children population, leads to significant adverse direct and indirect impacts on the gross domestic product [GDP) [5,6].

Thus far, strain-specific immunization is the most effective strategy to prevent influenza infections. Vulnerable individuals such as elderly individuals, pregnant women, and health care workers are the primary target of common influenza vaccination programs [7,8]. The extension of the immunization offered to children and adolescents (2–18 years old) in the U.S. with the use of intranasal live-attenuated influenza vaccines (LAIVs), resulting in 20–25% coverage, has showed a reduction in pediatric hospitalizations and medical visits among adults [9,10]. Although children have the highest influenza incidence across age groups, who are also believed to be the major transmitters, the recommendation of pediatric influenza immunization is still underrepresented [11,12]. Free-of-charge vaccination is recommended for healthy children in many European countries such as Finland and Ireland [13,14].

One of the major considerations to prioritize a universal influenza vaccination program for healthy children is the economic impact of such a massive prevention strategy. Several modeling studies have reported the clinical, public health, and economic benefits of pediatric immunization against influenza with live-attenuated vaccines [15,16,17,18]. Economic evaluations in the U.S. and Canada have demonstrated that childhood vaccination was very likely cost-effective or cost-saving [19,20,21,22,23]. This systematic review aimed to study the cost-effectiveness profile of LAIVs in pediatric vaccination programs against no vaccination or status quo immunization strategies. The findings of this study will provide crucial information for health policymakers to evaluate the implementation of pediatric influenza vaccination with LAIV and whether this should be considered as a routine preventive strategy among children.

## 2. Methods

### 2.1. Inclusion and Exclusion Criteria

We included published modeling studies that assessed the cost-effectiveness of intranasal LAIV in the pediatric population (aged 18 years or below) without specific health conditions or coexisting diseases. Studies that adopted full-scale decision analytical models to evaluate the economic outcomes of the pediatric vaccination program were also included. Studies with the comparators including the current vaccination strategy, no vaccination, or vaccination with other vaccine types were either eligible. Only full-text original articles published in English were retrieved and evaluated for inclusion in this systematic review.

The exclusion criteria were as follows: (1) health effects were not reported in quality-adjusted life years (QALYs), disability-adjusted life years (DALYs) or life years (LYs); (2) incremental cost-effectiveness ratio (ICER) was absent; and (3) publications were editorials, letters, commentaries or conference abstracts.

### 2.2. Literature Search and Study Selection

In line with the Preferred Reporting Items for Systematic Review and Meta-Analysis (PRISMA) guidelines, we conducted a systematic literature search using Pub-Med/MEDLINE, Embase, and Scopus, covering the period from 1 January 2000 to 30 April 2022 in light of the rapid changes in the epidemiology of influenza and the recommendation and availability of new influenza vaccines. The search terms were as follows: (“nasal” OR “intranasal” OR “Live attenuated” OR “LAIV”) AND “influenza vaccin*” AND (“Health Economic” OR “cost-effectiveness” OR “cost-benefit”) AND (“children” OR “school” OR “pediatric”). Titles and abstracts were screened by two authors (SC and HC), and the full text of potentially eligible articles was assessed for final inclusion. Manual searches through the reference lists of relevant studies were also performed for additional articles. Our review was also registered in PROSPERO (ID 343393).

### 2.3. Data Extraction

The titles and abstracts based on the search from the electronic databases were screened after removing duplications to check for eligibility by two authors (SC and HC). The full text of the potentially eligible publications was reviewed. In the case of disagreement, another author was consulted (CW). The reported data for any relevant variable were extracted by two authors (SC and HC). These included the year of publication, country, setting, perspective, target population, vaccination strategy, comparator, vaccine coverage, type of vaccine, type of mathematical model, time horizon, discount rate, willingness to pay (WTP) threshold use, and economic outcomes (ICERs in terms of cost per gain in QALY, DALY, or LY). All monetary values were converted to U.S. dollars.

### 2.4. Quality Assessment

The Consensus Health Economic Criteria (CHEC) Extended Checklist was used by two authors (SC and HC) to assess the methodological quality of reporting the cost-effectiveness of childhood vaccination with LAIV in the included studies [24]. Twenty items were included in the CHEC Extended Checklist, in which positive responses were scored 1, while negative responses were scored 0. The quality of each study was categorized as low, moderate, good, or excellent by its total CHEC score. The score for each item was summed, and the total CHEC score was transformed to a percentage from 0% to 100%. The quality of the study was classified into “low”, “moderate”, “good”, and “excellent” by cutoffs of <50%, 51–75%, 76–95%, and >95%, respectively. Higher scores denote a lower risk of bias. A third author (CW) resolved the differences in opinions.

## 3. Results

### 3.1. Study Characteristics

**Figure 1:**
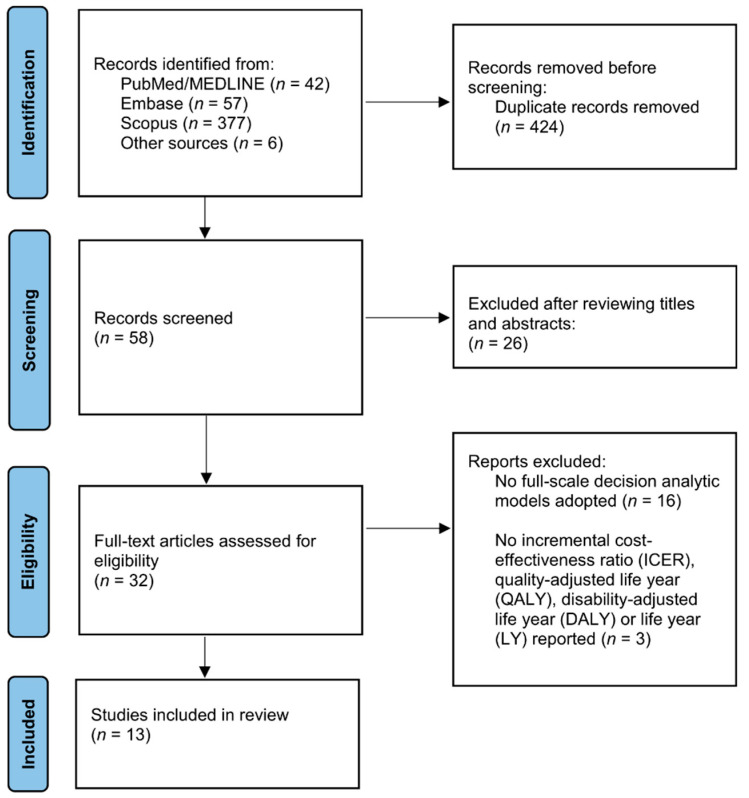
The literature search flow diagram.

We identified 58 records from the initial screening of titles and abstracts and reviewed 32 reports in full-text (Figure 1). Thirteen studies were finally deemed eligible for inclusion [19,20,21,23,25,26,27,28,29,30,31,32]. One study evaluated the LAIV vaccination in four countries [28]. Three studies were the economic evaluations of childhood vaccination against influenza in the U.S., and another five studied in England and/or Wales [20,21,23,25,26,28,30,32]. The other studies covered countries in Canada, Germany, France, the Netherlands, Spain, Finland, Brazil, and Taiwan [19,20,27,28,29,31]. 

Table 1 summarizes the main characteristics of the included economic evaluations. The target population of immunization among most of the studies included children aged 2–17 years. For instance, the immunization program was foreseen for children aged 2–8 and 2–16 years in the studies by Shim et al. and Baguelin et al., respectively [20,30]. Enlarged scenarios were also hypothesized by Thorrington et al. (2–11 and 2–17 years), Pitman et al. (2–4, 2–10 and 2–18 years), and de Boer et al. (2–6, 2–12 and 2–17 years) [24,26,32]. Wenzel et al. analyzed the immunization strategies for children aged 2–4, 5–11, and 12–16 years separately [25]. In addition, children at 6 months were included in two studies [21,23].

In 10 studies, the addition of the childhood vaccination with LAIVs to the influenza immunization program was compared to the status quo immunization strategies such as vaccination to the elderly and other high-risk groups with trivalent inactivated vaccine (TIV) (Table 1) [24,25,27,28,30,31,32]. Five studies evaluated the use of LAIVs against no vaccination or the current use of TIV in children [19,20,21,23,29]. Thorrington et al. evaluated the cost-effectiveness of quadrivalent LAIVs over trivalent LAIVs [26]. The majority of studies assumed the efficacy of LAIV to be from 70% to 80%. De Boer et al. adopted a lower value for LAIV (45%), while Thorrington et al. applied different efficacy values against influenza A and B [24,26]. Fifty percent vaccination coverage was usually assumed in the childhood vaccination program among the studies included (Table 2) [24,27,28,30,31,32].

The dynamic transmission model was the most commonly adopted method to evaluate the cost-effectiveness of childhood influenza vaccination (*n* = 10) [20,24,25,26,27,28,29,30,31,32] (Table 1). The remaining evaluations used the decision-tree model [19,21,23]. Eleven comparisons were evaluated from the payer perspective or public sector perspective [20,21,25,26,28,30,31,32]. One study undertook an evaluation from a societal perspective [23]. The remaining studies analyzed the findings from the perspectives of both the payer and society [19,24,27,29]. Four evaluations set the time horizon ranging from 10 months to 2 years [19,20,21,25], and five evaluations reported the findings in 5 years [23,28]. Other publications considered longer time horizons of 10–30 years or even of 200 years [24,26,27,29,30,32].

Table 3 summarizes the quality assessment for each study included in this systematic review. All studies were of good or excellent quality, indicating a lower risk of bias. Most of the included studies (61.5%) received financial support from pharmaceutical companies, while four studies were funded by public health care organizations. One study did not report the funding sources or conflicts of interest [25].

### 3.2. Economic Evaluations

The economic data of the studies are presented in Table 2. The costs of the vaccines varied from $3.84 to $32.3 (per dose) for LAIV and from $3.8 to $14.6 (per dose) for inactivated vaccines. Three evaluations assumed the same cost for both the LAIV and inactivated vaccines (21,23,24]. In terms of the discount rate, seven, five, and two comparisons examined it at 3%, 3.5%, and 4%, respectively, while two studies did not specify the discount rate [19,21]. Fifteen evaluations calculated the ICER as cost per QALY gained, while the ICER was calculated as the cost/DALY and cost/LY in two separate studies (Table 2). All evaluations reported the WTP thresholds and all performed one-way, two-way, or probabilistic sensitivity analyses.

**Figure 2:**
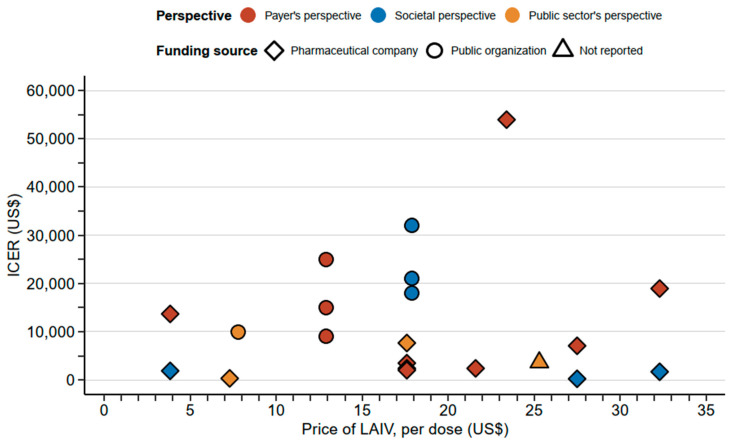
Incremental cost-effectiveness ratios against vaccine prices. Abbreviations: ICER—increment cost-effectiveness ratio; LAIV—live-attenuated influenza vaccine.

Compared with the population-based influenza immunization only for the elderly and high-risk groups or no vaccination for the pediatric population, the addition of childhood vaccination with intranasal LAIV offers an overall cost-effective strategy [21,23,24,25,27,28,29,30,31,32]. Regarding the base-case scenarios considered in the studies, most studies reported ICERs below $20,000 per unit change in QALY, DALY, or LY regardless of the prices of the LAIVs considered (Figure 2). Shim et al. set the price of LAIVs at $23.4 per dose and estimated an ICER of $53,960/QALY, which was the highest estimated ICER among the studies. Prosser et al. demonstrated the cost-effectiveness of LAIV in a younger age group (6 months to 2 years) [21,23]. The cost-effectiveness of pediatric influenza vaccination remains, even with the use of quadrivalent LAIV, which is more expensive but substantially increases the clinical impacts and health benefits for the pediatric population [26]. The sensitivity analyses within all of the included studies demonstrated the robustness of the cost-effectiveness of the pediatric influenza vaccination. The most common influencing parameters were the efficacy of LAIV among children, vaccination coverage, and the vaccine price. Two studies also included the indirect protection and thus health benefits arising from herd immunity to the rest of the population in addition to the target group [24,32]. The cost-effectiveness of pediatric vaccination with LAIV was demonstrated with the inclusion of herd immunity.

## 4. Discussion

The public health burden of seasonal influenza is significant, as demonstrated by the number of incident cases, hospitalizations, and deaths [1,2,3,4]. Influenza immunization is the most effective strategy to prevent infections and subsequent severe complications, and annual vaccination for priority groups is recommended by the World Health Organization [7,8]. Influenza incidence is age-dependent, and a significantly higher incidence rate is observed in the pediatric population [5,6]. Possible reasons include the immaturity of their immune systems and their social interactions in settings such as attendance at schools [5,6,33,34]. Furthermore, the duration of influenza viral shedding in children is longer than that in adults [33,34]. These findings contribute to the rationale of extending the influenza vaccination to the pediatric population, which is in place in some countries [35,36]. Positive outcomes are highlighted in countries where vaccination with LAIV is extended to a younger population, as demonstrated by the reduction in influenza cases, medical visits, and hospitalizations in this age group. Other than the health benefits, economic impact is another major factor in the consideration of universal vaccination against influenza in the pediatric population. 

This systematic review, to our knowledge, is the first to summarize the cost-effectiveness of vaccination with LAIV in children with a wide-ranging and up-to-date body of data. First, our findings indicate that the implementation of influenza vaccination with intranasal LAIV in the pediatric population was cost-effective, regardless of the perspectives or comparisons with the immunization strategy for the elderly and the high-risk groups alone or with no vaccination. Second, the efficacy of LAIV for children, vaccination coverage, and the vaccine price were the significant factors that contributed to the cost-effectiveness of the influenza vaccination for children. Third, herd immunity arising from pediatric immunization with LAIV provided an additional value to the strategy, given the frequent school activities among children.

The efficacy and price of LAIVs are the key parameters to the cost-effectiveness of pediatric influenza vaccination. Previous meta-analyses have demonstrated high LAIV efficacy ranging from 72% to 80% among children and that its efficacy against the matched strains was even higher [17,37,38]. This could be explained by the more effective stimulation of the immune response in light of the nature of intranasal administration and the infection of LAIV [39,40]. The relatively high efficacy of LAIV, however, is not observed in adults [41]. This further underlines the potential difference in the vaccination strategy between age groups. Future techniques in vaccine manufacturing may be required to further increase the efficacy [42]. The health benefits of quadrivalent LAIV over the conventional trivalent LAIV are also an additional consideration in future cost-effectiveness analysis and policy implementation [26]. The vaccine price and affordability should be considered when developing vaccination strategies. Financial burden arising from the pediatric influenza vaccination in addition to the existing strategy may be worrisome for low- or middle-income regions in particular, given that mass influenza immunization programs may incur high costs annually. While the studies in our review were mostly conducted in high-income countries or regions, further full-scale economic investigations are required in the settings of developing economies with low or middle income.

Another driver of the cost-effectiveness of the pediatric influenza vaccination is vaccination coverage. Compared with the injection of the inactivated influenza vaccine, which is commonly linked to pain and other disease transmissions, intranasal LAIV is noninvasive with lowered discomfort, which improves the acceptance of the children and parents, and thus the coverage of the influenza vaccination [43]. Most of the studies in this review assumed the vaccination coverage to be nearly 50%, while coverage in real-world settings may be lower, for example, 26% among younger children in China [44,45]. The low uptake of the influenza vaccine in the pediatric population may be explained by the parents’ perceived severity of influenza, perceived safety, and efficacy of the vaccine [44]. The outcomes of the cost-effectiveness analysis should therefore be interpreted with caution, and further efforts should be made to explore and address the root problems for higher coverage.

The included studies in this review highlighted the importance of herd immunity on the cost-effectiveness of the pediatric immunization program with LAIV [24,32]. In addition to the direct health benefits of LAIV for the children who are vaccinated, childhood influenza vaccination offers indirect protection for the rest of the population including but not limited to their peers and family members, adults, and other vulnerable groups in the wider community. In Japan, childhood influenza vaccination has been mandatory for more than a decade since the 1960s, and the excess mortality rate in the subsequent years from influenza and pneumonia has dropped since the initiation of the pediatric influenza program [46,47]. However, the laws were relaxed in the mid-1980s due to the controversy over vaccine efficacy. The vaccine coverage in schoolchildren was notably reduced, resulting in a significant increase in excess deaths from influenza and pneumonia in the general population [46,47]. The Japanese experience suggests that herd immunity arising from pediatric influenza vaccination would provide indirect protection to the neighboring community, especially elderly individuals. With the mandatory influenza vaccination among the school children from the 1960s to early 1980s, the mortality from influenza in the elderly was reduced by approximately 40% [45]. The impact of herd community on an unvaccinated vulnerable population may possibly promote the cost-effectiveness of the influenza vaccination with LAIVs in the pediatric population. Further studies may be conducted in the future to investigate such potential economic values. 

Nevertheless, this study had a few drawbacks, although we summarized a wide-ranging body of evidence with the most recently updated results. First, our study may be limited by the heterogeneity of the included studies. The differences in areas, for example, age groups, time horizons, discount rates, and perspectives, may compromise the comparability of the studies in this review. Second, studies were selected based on the full-text availability with the inclusion of the entire pediatric population. Indeed, the economic benefits of pediatric influenza immunization may vary for specific target subgroups such as children with asthma. Further studies are warranted in the future to summarize the evidence of the cost-effectiveness of immunization programs for children with specific medical conditions. Our restriction to studies with the use of a single economic measure (ICER expressed in cost per QALY or LY gained) for a fair comparison may limit the scope of evidence in this area. In light of a more comprehensive economic evaluation of pediatric influenza vaccination, further studies may be conducted to capture other standardized measures, for instance, cost-saving and budget impacts.

## 5. Conclusions

The implementation of influenza vaccination in the pediatric population with LAIV is likely to be cost-effective. The efficacy of LAIV for children, vaccination coverage, and the vaccine price are significant factors in the consideration of the cost-effectiveness of an influenza vaccine for children. Herd immunity arising from pediatric immunization against influenza provides additional value to this preventive strategy. This systematic review thus offers important insights for policymakers and health authorities on planning and developing the pediatric influenza vaccination program in different countries and regions.

## Figures and Tables

**Table 1:** The characteristics of the included studies.

Study	Setting	Year	Model Type	Population	Vaccination Strategy	Comparator	Perspective	Time Horizon	Discount Rate	Sensitivity Analysis	Funding Source
De Boer [24]	The Netherlands	2021	Dynamic transmission model	Children 2–16 Y	LAIV (in addition to the current strategy	Current strategy (people at risk and the elderly with TIV)	Societal; payer’s	20 years	4%	One way and probabilistic sensitivity analysis	Vaccine manufacturer
Wenzel [25]	England, Wales	2021	Dynamic transmission model	Pre-school (2–4 Y); Primary School (5–11 Y); Secondary school (12–16 Y) All children (2–16 Y)	LAIV (in addition to the current strategy)	Current strategy (people at risk and the elderly with TIV)	Public sector	1 year	3.5%	One way and probabilistic sensitivity analysis	NR
Thorrington [26]	England	2017	Dynamic transmission model	Children 2–11 Y; 2–16 Y	Quadrivalent LAIV for children and quadrivalent IIV for people at risk and the elderly	Current strategy (children with trivalent LAIV and people at risk and the elderly with TIV)	Public sector	14 years	3.5%	One way and probabilistic sensitivity analysis	NHS
Gerlier [27]	France	2016	Dynamic transmission model	Children 2–17 Y	LAIV (in addition to the current strategy)	Current strategy (people at risk and the elderly with TIV)	Societal; payer’s	30 years	4%	One way and probabilistic sensitivity analysis	Vaccine manufacturer
Gibson [28]	England, Wales	2016	Dynamic transmission model	Children 2–17 Y	LAIV (in addition to the current strategy)	Current strategy (people at risk and the elderly with TIV)	Public sector	5 years	3.5%	One way and probabilistic sensitivity analysis	Vaccine manufacturer
Gibson [28]	Brazil	2016	Dynamic transmission model	Children 2–17 Y	LAIV (in addition to the current strategy)	Current strategy (people at risk and the elderly with TIV)	Payer’s	5 years	3%	One way and probabilistic sensitivity analysis	Vaccine manufacturer
Gibson [28]	Spain	2016	Dynamic transmission model	Children 2–17 Y	LAIV (in addition to the current strategy)	Current strategy (people at risk and the elderly with TIV)	Payer’s	5 years	3%	One way and probabilistic sensitivity analysis	Vaccine manufacturer
Gibson [28]	Taiwan	2016	Dynamic transmission model	Children 2–17 Y	LAIV (in addition to the current strategy)	Current strategy (people at risk and the elderly with TIV)	Payer’s	5 years	3%	One way and probabilistic sensitivity analysis	Vaccine manufacturer
Nagy [29]	Finland	2016	Dynamic transmission model	Children 2–17 Y	LAIV	No vaccination among children	Societal; payer’s	20 years	3%	One way and probabilistic sensitivity analysis	Vaccine manufacturer
Shim [20]	USA	2016	Dynamic transmission model	Children 2–8 Y	LAIV	TIV among children	Public sector’s	10 months	3%	Two way and probabilistic sensitivity analysis	Vaccine manufacturer
Baguelin [30]	England, Wales	2015	Dynamic transmission model	Children 2–16 Y	LAIV (in addition to the current strategy)	Current strategy (the elderly with TIV)	Public sector	14 years	3.5%	One way and probabilistic sensitivity analysis	NIHR
Damm [31]	Germany	2015	Dynamic transmission model	Children 2–17 Y	LAIV (in addition to the current strategy)	Current strategy (people at risk with TIV)	Narrow third-party payer; board third-party payer	14-year run-in phase; 10-year intervention	3%	One way and probabilistic sensitivity analysis	Vaccine manufacturer
Pitman [32]	England, Wales	2013	Dynamic transmission model	Children 2–4 Y; 2–10 Y; 2–18 Y	LAIV (in addition to the current strategy)	Current strategy (the elderly with TIV)	Public sector	200 years	3.5%	One way and probabilistic sensitivity analysis	Vaccine manufacturer
Tarride [19]	Canada	2012	Decision-tree model	Children 2–17 Y	LAIV	Current use of TIV among children	Societal; payer’s	1 year	NR	One way and probabilistic sensitivity analysis	Vaccine manufacturer
Prosser [23]	USA	2011	Decision-tree model	Children 6 M–23 M; 2 Y; 3–4 Y	LAIV	No vaccination among children	Societal	5 years	3%	One way and probabilistic sensitivity analysis	CDC
Prosser [21]	USA	2006	Decision-tree model	Children 6 M–23 M; 2 Y; 3–4 Y; 5–11 Y; 12–17 Y	LAIV	No vaccination among children	Payer’s	1 year	NR	One way, two way and probabilistic sensitivity analysis	CDC

Abbreviations: CDC—Centers for Disease Control and Prevention; IIV—inactivated influenza vaccine; LAIV—live-attenuated influenza vaccine; M—months; NHS—National Health Service; NIHR—National Institute for Health and Care Research; NR—not reported; TIV—trivalent inactivated vaccine; Y—years.

**Table 2:** A summary of the results of the included studies.

Study	Vaccine Price (per Dose)	Vaccine Coverage	LAIV Efficacy	WTP Threshold	Outcomes	Conclusions
De Boer [24]	LAIV: $3.84 TIV: $3.84	50%	48%	$21,040 (€20,000)	Societal perspective: ICER = $1868/QALY Payer’s perspective: ICER = $13,680/QALY	Vaccinating 2–16 Y with LAIV in addition to the current strategy is cost-effective.
Wenzel [25]	LAIV: $25.3	55%	70%	$24,632 (£20,000)	Pre-school (2–4 Y): ICER = $2530/QALY Primary School (5–11 Y): ICER = $787/QALY Secondary school (12–16 Y): ICER = $27,930/QALY All children (2–16 Y): ICER = $3583/QALY	Vaccinating 2–16 Y with LAIV in addition to the current strategy is cost-effective. Vaccinating primary school students (5–11 Y) is the most cost-effective.
Thorrington [26]	NR	<5 Y: 33.7% 5–16 Y: 54.9% High risk group: 45.1% Elderly: 71.0%	70%	$24,632 (£20,000)	Maximum incremental cost of the quadrivalent vaccine = $0.25	Quadrivalent influenza vaccines are cost-effective.
Gerlier [27]	LAIV: $32.3 TIV: $6.4	50%	80%	$32,612 (€31,000)	Societal perspective: ICER = $1679/LY Payer’s perspective: ICER = $18,937/LY	Vaccinating 2–17 Y with LAIV in addition to the current strategy is cost-effective.
Gibson [28]	LAIV: $17.6 TIV: $7.9	50%	80%	$31,560 (£30,000)	ICER = $6531/QALY	Vaccinating 2–17 Y with LAIV in addition to the current strategy is cost-effective in England and Wales
Gibson [28]	LAIV: $17.6 TIV: $5.0	50%	80%	$31,560 (£30,000)	ICER = $2963/QALY	Vaccinating 2–17 Y with LAIV in addition to the current strategy is cost-effective in Brazil
Gibson [28]	LAIV: $17.6 TIV: $2.8	50%	80%	$31,560 (£30,000)	ICER = $2022/QALY	Vaccinating 2–17 Y with LAIV in addition to the current strategy is cost-effective in Spain
Gibson [28]	LAIV: $17.6 TIV: $4.5	50%	80%	$31,560 (£30,000)	ICER = $1708/QALY	Vaccinating 2–17 Y with LAIV in addition to the current strategy is cost-effective in Taiwan
Nagy [29]	LAIV: $27.5	22–28%	80%	$6312 (€6000)	Societal perspective: ICER = $189/QALY Payer’s perspective: ICER = $6032/QALY	Vaccinating 2–17 Y with LAIV is cost-effective
Shim [20]	LAIV: $23.4 TIV: $14.6	58%	83%	$159,123	Public sector’s perspective: ICER = $53,960/QALY	Vaccinating 2–8 Y with LAIV is cost-effective over TIV.
Baguelin [30]	LAIV: $7.8 TIV: $7.8	50%	70%	$30,791 (£25,000)	ICER: $9968/QALY	Vaccinating 2–16 Y with LAIV in addition to the current strategy is cost-effective.
Damm [31]	LAIV: $21.6 TIV: $11.4	50%	80%	$52,600 (€50,000)	Narrow third-party payer: ICER = $2507/QALY Board third-party payer: ICER = $1359/QALY	Vaccinating 2–16 Y with LAIV in addition to the current strategy is cost-effective.
Pitman [32]	LAIV: $7.3 TIV: $7.3	50%	80%	$24,632 (£20,000)	ICER: $309/QALY	Vaccinating 2–18 Y with LAIV in addition to the current strategy is cost-effective.
Tarride [19]	LAIV: $11.1 TIV: $7.6	37% (2–5 Y); 42% (6–9 Y); 100% (10–17 Y)	60%	$39,152 (CAD$50,000)	Societal perspective: LAIV is the dominant strategy Payer’s perspective: LAIV is the dominant strategy	Vaccinating 2–16 Y with LAIV is cost-effective over TIV.
Prosser [23]	LAIV: $17.9	NR	84%	$150,000	6 M–23 M: $18,000/QALY 2 Y: $21,000/QALY 3–4 Y: $32,000/QALY	Vaccinating with LAIV is cost-effective for different age groups. Cost-effectiveness among children decreases with increasing age.
Prosser [21]	LAIV: $12.9	NR	84%	$150,000	6 M–23 M: $9000/QALY 2 Y: $15,000/QALY 3–4 Y: $25,000/QALY 5–11 Y: $72,000/QALY 12–17 Y: $109,000/QALY	Vaccinating with LAIV is cost-effective for different age groups. Cost-effectiveness decreases (i.e., higher ICER) when increasing the vaccination age among children.

All monetary values are expressed in US dollars. ICER—incremental cost-effectiveness ratio; LAIV—live-attenuated influenza vaccine; LY—life years; M—months; NR—not reported; QALY—quality-adjusted life years; TIV—trivalent inactivated vaccine; WTP—willingness to pay; Y—years.

**Table 3:** The quality assessment of the studies included.

Study No.	Checklist Question	De Boer 2021 [24]	Wenzel 2011 [25]	Thorrington 2017 [26]	Gerlier 2016 [27]	Gibson 2016 * [28]	Nagy 2016 [29]	Shim 2016 [30]	Baguelin 2015 [31]	Damm 2015 [32]	Pitman 2013 [33]	Tarride 2012 [19]	Prosser 2011 [23]	Prosser 2006 [21]	Total (% of Yes)
1	Is the study population clearly described?	1	1	1	1	1	1	1	1	1	1	1	1	1	100
2	Are competing alternatives clearly described?	1	1	1	1	1	1	1	1	1	1	1	1	1	100
3	Is a well-defined research question posed in answerable form?	1	1	1	1	1	1	1	1	1	1	1	1	1	100
4	Is the economic study design appropriate to the stated objective?	1	1	1	1	1	1	1	1	1	1	1	1	1	100
5	Are the structural assumptions and the validation methods of the model properly reported?	1	1	1	1	1	1	1	1	1	1	1	1	1	100
6	Is the chosen time horizon appropriate in order to include the relevant costs and consequences?	1	1	1	1	1	1	1	1	1	1	1	1	1	100
7	Is the actual perspective chosen appropriate?	1	1	1	1	1	1	1	1	1	1	1	1	1	100
8	Are all important and relevant costs for each alternative identified?	1	1	1	1	0	0	1	1	1	1	1	1	1	84.6
9	Are all costs measured appropriately in physical units?	1	1	1	1	1	1	1	1	1	1	1	1	1	100
10	Are costs valued appropriately?	1	1	1	1	1	1	1	1	1	1	1	1	1	100
11	Are all important and relevant outcomes for each alternative identified?	1	1	1	1	1	1	1	1	1	1	1	1	1	100
12	Are all outcomes measured appropriately?	1	1	1	1	1	1	1	1	1	1	1	1	1	100
13	Are outcomes valued appropriately?	1	1	1	1	1	1	1	1	1	1	1	1	1	100
14	Is an appropriate incremental analysis of costs and outcomes of alternatives performed?	1	1	1	1	1	1	1	1	1	1	1	1	1	100
15	Are all future costs and outcomes discounted appropriately?	1	1	1	1	1	1	1	1	1	1	0	1	0	84.6
16	Are all important variables, whose values are uncertain, appropriately subjected to sensitivity analysis?	1	1	1	1	1	1	1	1	1	1	1	1	1	100
17	Do the conclusions follow from the data reported?	1	1	1	1	1	1	1	1	1	1	1	1	1	100
18	Does the study discuss the generalizability of the results to other settings and patient/client groups?	0	0	0	0	1	0	0	0	0	1	1	1	1	38.4
19	Does the article/report indicate that there is no potential conflict of interest of study researcher(s) and funder(s)?	1	0	1	1	1	1	1	1	1	0	1	1	1	84.6
20	Are ethical and distributional issues discussed appropriately?	0	0	1	1	1	1	0	0	1	0	0	0	0	38.4
	% of Yes	90	85	100	95	95	90	90	90	95	90	90	95	90	
	Overall quality	Good	Good	Excellent	Good	Good	Good	Good	Good	Good	Good	Good	Good	Good	

The CHEC extended checklist consists of 20 items with positive responses scored 1 and negative responses scored 0. The total score for each item was summed and converted to a percentage with the range of scores ranging from zero to 100. The total CHEC score for each study was categorized into four grades: low, moderate, good, and excellent using cut-off value of <50, 51–75, 76–95, and >95, respectively. Higher scores denote a lower risk of bias. * There were four comparisons in the study of Gibson [28].

## Data Availability

No new data were created or analyzed in this study. Data sharing is not applicable to this article.
